# Enhanced immunosuppressive capability of mesenchymal stem cell-derived small extracellular vesicles with high expression of CD73 in experimental autoimmune uveitis

**DOI:** 10.1186/s13287-024-03764-7

**Published:** 2024-05-23

**Authors:** Yanan Duan, Xiteng Chen, Hui Shao, Yongtao Li, Zhihui Zhang, Huan Li, Chuan Zhao, Hong Xiao, Jiawei Wang, Xiaomin Zhang

**Affiliations:** 1 Tianjin Key Laboratory of Retinal Functions and Diseases, Tianjin Branch of National Clinical Research Center for Ocular Disease, Eye Institute and School of Optometry, Tianjin Medical University Eye Hospital, Tianjin, China; 2 Department of Ophthalmology and Visual Sciences, Kentucky Lions Eye Center, School of Medicine, University of Louisville, Louisville, KY USA

**Keywords:** Mesenchymal stem cell, Small extracellular vesicle, CD73, Adenosine, Autoimmune uveitis

## Abstract

**Background:**

Autoimmune uveitis is an inflammatory disease triggered by an aberrant immune response. Mesenchymal stem cell-derived small extracellular vesicles (MSC-sEVs) are emerging as potential therapeutic agents for this condition. CD73, an ectoenzyme present on MSC-sEVs, is involved in mitigating inflammation by converting extracellular adenosine monophosphate into adenosine. We hypothesize that the inhibitory effect of MSC-sEVs on experimental autoimmune uveitis (EAU) could be partially attributed to the surface expression of CD73.

**Methods:**

To investigate novel therapeutic approaches for autoimmune uveitis, we performed lentiviral transduction to overexpress CD73 on the surface of MSC-sEVs, yielding CD73-enriched MSC-sEVs (sEVs-CD73). Mice with interphotoreceptor retinoid-binding protein (IRBP)-induced EAU were grouped randomly and treated with 50 µg MSC-sEVs, vector infected MSC-sEVs, sEVs-CD73 or PBS via single tail vein injection. We evaluated the clinical and histological features of the induced mice and analyzed the proportion and functional capabilities of T helper cells. Furthermore, T-cells were co-cultured with various MSC-sEVs in vitro, and we quantified the resulting inflammatory response to assess the potential therapeutic benefits of sEVs-CD73.

**Results:**

Compared to MSC-sEVs, sEVs-CD73 significantly alleviates EAU, leading to reduced inflammation and diminished tissue damage. Treatment with sEVs-CD73 results in a decreased proportion of Th1 cells in the spleen, draining lymph nodes, and eyes, accompanied by an increased proportion of regulatory T-cells (Treg cells). In vitro assays further reveal that sEVs-CD73 inhibits T-cell proliferation, suppresses Th1 cells differentiation, and enhances Treg cells proportion.

**Conclusion:**

Over-expression of CD73 on MSC-sEVs enhances their immunosuppressive effects in EAU, indicating that sEVs-CD73 has the potential as an efficient immunotherapeutic agent for autoimmune uveitis.

**Supplementary Information:**

The online version contains supplementary material available at 10.1186/s13287-024-03764-7.

## Background

Autoimmune uveitis is a complex and heterogenous inflammation that manifests with diverse symptoms, including blurred vision, photophobia and pain [1, 2]. Several therapeutic modalities, such as glucocorticoids (GCs) and immunosuppressive agents, are employed to address the intricacies of this condition. Predominantly, GCs stand as the primary choice in clinical uveitis treatment. However, their protracted usage is encumbered by drawbacks, which encompass adverse effects like cataracts, glaucoma, and systemic repercussions [3–5]. Other methodologies also exhibit certain limitations. Consequently, ongoing researches into novel therapies aspire to not only heighten treatment efficacy but also mitigate adverse effects, ultimately refining the comprehensive management of uveitis.

In recent years, some studies have assessed both the efficacy and safety of mesenchymal stem cells (MSCs) in addressing experimental autoimmune uveitis (EAU) in animal models [6–8]. MSCs, widely recognized for their regenerative and immunomodulatory functions, have been utilized in various neurological and immune disorders, including autoimmune diseases [9, 10]. Recent studies also indicate their significant role in COVID-19 patients, particularly in virus-host interaction, drug testing, tissue regeneration, and immune modulation [11]. Nonetheless, MSC-based therapy not only presents challenges in cell product preservation and transport, but also elevates the risks associated with vessel obstruction, malignant transformation, and allogenic immunological rejection [12–15]. To mitigate these obstacles, attention has turned to their secretions, small extracellular vesicles (sEVs). Following the recommendations of Minimal information for studies of extracellular vesicles (MISEV) [16], we define extracellular vesicles with diameters less than 200 nm as sEVs, rather than exosomes or ectosomes. These nano-sized vesicles play a pivotal role in shuttling nutrient substances to mediate recipient cell functions, mirroring the impact of their parent cells [17–19]. The lipid bilayer membrane structure of sEVs could protect their cargo from degradation. Consequently, they have been extensively harnessed as ideal drug carriers and strategically modified for therapeutic interventions in various immune disorders. Furthermore, the utilization of MSC-derived sEVs (MSC-sEVs) holds promise for therapeutic agent applications, as they offer improved safety and stability compared to their parent MSCs. Nevertheless, the current immunosuppressive potency of MSC-sEVs falls short for clinical implementation, and ongoing research endeavors are dedicated to optimizing the use of MSC-sEVs by exploring their immunomodulatory mechanism [19].

CD73, also known as ecto-5’-nucleotidase (5’-NT, eN, eNT, NT5E), plays a pivotal role in purinergic signaling [20]. It facilitates the conversion of extracellular pro-inflammatory adenosine triphosphate (ATP) into anti-inflammatory adenosine [21]. Adenosine acts on various immune cells by binding to adenosine receptors on the cell surface (A1, A2A, A2B, and A3) [22], influencing cellular function through modulation of intracellular cAMP levels. While adenosine receptors A1AR and A3AR reduce cAMP levels, A2AR and A2BAR elevate intracellular cAMP levels, leading to activation of protein kinase A (PKA) pathways and inhibition of NF-κB and JAK-STAT signaling pathways [23], thereby suppressing inflammation in lymphocyte-mediated immune responses [24–26]. As the activity of CD73 is irreversible, ongoing research on CD73 holds promise, particularly in its potential to modulate immune responses via adenosine production. Research on CD73 is currently predominantly focused on tumor immunology. CD73-produced adenosine suppresses anti-tumor immune responses and blocking CD73 shows potential as a cancer treatment strategy [27]. However, the intricacies of CD73 in immune regulation highlight its significance in autoimmune diseases. Chen et al. first reported that the surface CD73 of MSCs can catalyze the production of adenosine, which, upon binding to the A2A adenosine receptor, inhibits the proliferation of Th1 cells [28]. Lee et al. demonstrated that human bone marrow-derived MSCs exhibit robust immunosuppressive capabilities by modulating Th17 cell responses through the CD39-CD73-mediated pathway [29]. Further research has indicated that MSCs can indirectly dampen inflammation by increasing the proportion of regulatory T-cells (Treg cells) through the adenosine pathways [30]. These findings proposed a potential mechanism by which MSCs exert their immunomodulatory effects on EAU. Notably, Schneider et al. showed that the AMPase activity of CD73 is predominantly provided by CD8^+^ T-cells, particularly in EVs [31]. Considering that CD73 is also expressed on MSC-sEVs, attention has shifted to exploring the relationship between MSC-sEVs and the CD39-CD73-adenosine signaling pathway. Kerkela et al. reported that MSC-sEVs can catalyze the production of adenosine through their surface marker CD73, thereby inhibiting T cell proliferation [32]. Crain et al. further investigated that MSC-sEVs can suppress the proliferation of CD4^+^ T-cells in a concentration-dependent manner, and this effect can be mitigated by the addition of A2A receptor antagonists [33]. These findings suggested that the adenosine signaling pathway may play crucial roles in the immune suppression mediated by MSC-sEVs. Consequently, we hypothesized that overexpressing CD73 could enhance the immunosuppressive potential of MSC-sEVs by regulating the Th1/Th17/Treg cell balance. Building upon this premise, our team successfully constructed MSC-sEVs overexpressing CD73 (sEVs-CD73) through lentiviral transduction. We then evaluated the immunosuppressive efficacy of sEVs-CD73 in experimental autoimmune uveitis (EAU) models.

## Materials and methods

### Experimental design

All experiments were carried out in compliance with ARRIVE guidelines 2.0. Mice were randomly assigned to cages using a computer-generated randomization process. All measurements were standardized to ensure consistency and accuracy.

### Animals

We used a total of 120 mice in our study. Each experimental and control group comprised 6 mice, based on our previous experience. Detailed information regarding the allocation of mice to specific experimental conditions is provided in the Materials and methods section. All female C57BL/6 mice (7–8 weeks old), purchased from GemPharmatech Co., Ltd. (China), were housed under specific pathogen-free (SPF) conditions. All mice were included in the experiment only after being confirmed to be free of any fundus or systemic diseases. Animal care and experimentation were conducted in accordance with the Association for Research in Vision and Ophthalmology (ARVO) Statement. In this study, tribromoethanol was used for anesthetizing mice, and cervical dislocation was employed for euthanizing the mice. All animal procedures were approved by the Animal Care and Use Committee of Tianjin Medical University Eye Hospital (TMUEC).

### Culture and identification of mesenchymal stem cells

Human umbilical cord-derived MSCs were provided by Beijing Beilai Biological Co., Ltd. (China), and MSC isolation and culture were performed as previously described [34]. Various methods are available for the identification of MSCs, including flow cytometry and differentiation assays. According to International Society for Cell & Gene Therapy (ISCT), MSCs were identified by their capacity to express specific cell surface markers (CD73, CD90), and lack expression of hematopoietic lineage markers (CD34, CD45) [35]. Additionally, their ability to differentiate into adipocytes, chondrocytes, and osteocytes under defined conditions was demonstrated by staining in vitro.

### Production and transduction of Lentiviruses

Lentiviral vectors overexpressing CD73 and empty plasmids (pCDH-CMV-MCS-EF1-copGFP) (Hanbio Biotechnology, China) were packaged into human embryonic kidney 293 T cells (HEK-293 T) following the manufacturer’s instructions. The packaging plasmids and envelope plasmids were also involved in the transfection process. Lentiviral particles were harvested from the culture at 48 and 72 h post-transfection and subsequently concentrated by ultracentrifugation at 72,000×g for 2 h. The viral titer was assessed using the serial dilution method, as outlined in previous studies [36].

### Transfection and supernatant collection

When reaching approximately 60% confluency at passage 2, MSCs were exposed to a viral particle mixture with a multiplicity of infection (MOI) of 50 and 8 µg/ml polybrene (Sigma-Aldrich, USA) in the culture medium. Subsequently, we obtained normal MSCs (MSC-N), vector-infected MSCs (MSC-V), and CD73-overexpressed MSCs (MSC-CD73). The conditioned medium from the third to fifth passage was collected for the production of MSC-sEVs. MSCs were cultured with complete DMEM/F-12 (Gibco, USA) media containing 10% fetal bovine serum (FBS) (Gibco) and 100 U/mL penicillin and streptomycin (Gibco). Before use, FBS and DMEM/F-12 were diluted at a ratio of 1:4 and centrifuged at 11,000×g overnight at 4 °C. This procedure aimed to reduce potential experimental confounds associated with sEVs present in FBS.

### Quantitative real-time PCR (qRT-PCR)

The quantitative real-time PCR (qRT-PCR) was conducted to assess mRNA expression levels of different MSCs (MSC-N, MSC-V, MSC-CD73). Total RNA extraction was performed using the TRIzol reagent (Invitrogen, USA) following the manufacturer’s instructions. Subsequently, cDNA was synthesized utilizing the RevertAid First Strand cDNA Synthesis Kit (Thermo Fisher, USA). Each PCR reaction was set up in 384-well plates, comprising FastStart SYBR Green Master (Roche, Switzerland), cDNA, and 0.25 μm forward and reverse primers. The relative mRNA expression levels of the target genes were determined using the 2^−ΔΔCT^ method, with GAPDH serving as an internal standard [37]. The primer sequences used were as follows: CD73 forward: CCAGTACCAGGGCACTATCTG, reverse: TGGCTCGATCAGTCCTTCCA; GAPDH forward: AGGTCGGTGTGAACGGATTTG, reverse: GGGGTCGTTGATGGCAACA.

### Collection of mesenchymal stem cell-derived small extracellular vesicles

The supernatant from both uninfected and infected MSCs was collected after 48 h of incubation and separated by ultracentrifugation to obtain each group of MSC-sEVs. The separation process involved centrifugation at 200×g for 10 min at 4 °C, followed by centrifugation at 2000×g for 20 min at 4 °C, and then at 10,000×g for 30 min at 4 °C. Subsequently, the resulting supernatant underwent two additional rounds of centrifugation at 110,000×g for 70 min at 4 °C to achieve a high concentration of MSC-sEVs [38]. All ultracentrifugation steps were performed using an Optima XLA/I centrifuge equipped with an An-45Ti rotor (Beckman-Coulter, USA). The final pellets were resuspended in sterile PBS (Gibco), and the concentration was measured using a BCA protein assay kit (Solarbio, China).

### Identification of mesenchymal stem cell-derived small Extracellular vesicles

To accurately characterize and distinguish different types of MSC-sEVs, various techniques were employed in this study. Samples were quickly fixed with 50 µl 4% paraformaldehyde (Sigma-Aldrich) for 5 min and applied to carbon copper grids. Negative staining was then performed using 2% uranyl acetate solution (Sigma-Aldrich). After drying, MSC-sEVs were directly visualized using transmission electron microscopy (TEM). Additionally, nanoparticle tracking analysis (NTA) was used to measure the size and concentration of MSC-sEVs in a liquid medium. The particle size was analyzed using Nanosight (version 3.3). For identification of various protein markers associated with different MSC-sEVs, Western blot was used. The isolated MSC-sEVs were lysed to release their protein content, which was then separated by polyacrylamide gel electrophoresis (SDS-PAGE) based on size and charge. The separated proteins were transferred onto a polyvinylidene difluoride (PVDF) membrane (Sigma-Aldrich) and incubated with a 5% non-fat dried milk to prevent non-specific binding of the antibody. The membrane was then probed with antibodies specific to MSC-sEVs-associated proteins, including CD63 (Abcam), TSG-101 (Abcam), CD73 (Abcam) and β-actin (Abcam). Additionally, the expression of CD73 on the surface of MSC-sEVs was also identified using ELISA kits, following the manufacturer’s instructions (R&D Systems).

### Induction and treatment of experimental autoimmune uveitis

EAU in C57BL/6 mice was induced by immunization with an emulsion comprising equal volumes of complete Freund’s adjuvant (CFA, Sigma Aldrich) and 5 mg/mL desiccated Mycobacterium tuberculosis (TB, Sigma-Aldrich), as well as 300 µg interphotoreceptor retinoid-binding protein_651 − 670_ (IRBP_651 − 670_, LAQGAYRTAVDLESLASQLT) (Shanghai Hanhong, China) in PBS. This emulsion was then applied to four spots on the tail base and flank. In addition, mice were intraperitoneally administered 500 ng pertussis toxin (PTX) (List Biological Laboratories, USA) on the day of immunization and 24 h post-immunization. Immunized mice were randomly assigned to different groups using a computer-generated random number sequence. The groups included PBS, MSC-sEVs (sEVs-N), vector-infected MSC-sEVs (sEVs-V), and sEVs-CD73. On day 11 post immunization, different groups of mice were injected via tail vein with 50 µg diverse MSC-sEVs respectively or equal volume of PBS.

### Clinical and histological assessment of experimental autoimmune uveitis

EAU mice were examined every other day by head-mounted indirect fundoscopy from day 9 to day 21 post-immunization. On the 17th day post-immunization, mice were euthanized, and their eye tissues were fixed in 4% paraformaldehyde, paraffin-embedded, sectioned (4 μm), and stained with hematoxylin and eosin (H&E). The histopathological changes of retina were examined and scored. The incidence and severity of inflammation were assessed according to the criteria of Caspi [39].

### Optical coherence tomography

On the 17th day post-immunization, a total of six mice per group were anesthetized, and the pupils were dilated with 0.1% tropicamide. The mice were then placed in a prone position, and a corneal contact lens was used to stabilize the eye. Spectralis optical coherence tomography (OCT) (Heidelberg, Germany) was used to scan the retina, and the images were scored based on the criteria previously established by Gadjanski and colleagues [40].

### Flow Cytometry detection of inflammatory cells

Mice were sacrificed on day 17 after immunization and their eyeballs, spleens (SP) and lymph nodes (LNs) were separated and ground. To prepare single lymphocyte suspension, the spleen was lysed with red blood cell lysis buffer (Sigma), while the eyeball tissue was digested with 1 mg/ml of collagenase D (Sigma-Aldrich) for 1 h. The resulting cell suspension was filtered through a 70-µm filter and then centrifuged. Part of these cells were incubated in a 96-well plate with 50 ng/mL phorbol 12-myristate 13-acetate (Sigma), 1 µg/mL ionomycin (Sigma), and 1 µg/mL brefeldin A (Abcam, USA). After incubation of 4.5 h, these cells were utilized to assess the ratio of Th1 and Th17 cells, while the remaining cells were prepared into single cell suspensions to determine the proportion of Treg cells.

The cells were incubated with Brilliant Violet™ 711 anti-mouse CD4 antibody for 30 min at 4 °C, followed by fixation and permeabilization according to the manufacturer’s instructions. Subsequently, FITC anti-mouse IFN-γ and PE anti-mouse IL-17 A antibodies were used to stain Th1 and Th17 cells, respectively. For Treg cells, the cells were stained with FITC anti-CD25 and PE anti-FOXP3 antibodies. The proportion of IFN-γ, IL-17 A, CD25, and FOXP-3 was then assessed using a FACSCalibur flow cytometer (BD Biosciences, USA) and analyzed with FlowJo software (USA). The laser for Brilliant Violet™ 711 was set at 405 nm, while for PE and FITC, it was set at 488 nm. The fluorescence channels corresponded to 710/50 (BV711), 575/25 (PE), and 530/30 (FITC), respectively. All antibodies were purchased from BioLegend.

### Assay of T-cell proliferation in vitro by Carboxyfluorescein Diacetate Succinimidyl Ester (CFSE)

The 96-well plates were precoated with 10 µg/mL anti-mouse CD3 mAb (BioLegend) and 5 µg/mL anti-mouse CD28 mAb (BioLegend) to stimulate CD4^+^T-cells. The total CD4^+^T-cells were isolated from spleens of naive mice by positive CD4^+^T-cell isolation kit (Miltenyi Biotec, USA). The cells were then labeled with 1 µM carboxyfluorescein diacetate succinimidyl ester (CFSE) (Invitrogen) for 10 min and co-cultured with MSC-sEVs at a concentration of 10 µg/ml. After 72 h of incubation, the CFSE fluorescence intensity was measured by FACS and analyzed by FlowJo.

### T-cell differentiation assays in vitro

Naive CD4^+^T-cells from spleens of naive mice were isolated using a positive isolation kit (Miltenyi Biotec). These purified cells were seeded at a density of 2 × 10^5^ cells/well in 96-well plates pre-coated with anti-CD3/CD28 and then cultured under Th1, Th17, and Treg differentiation conditions, respectively. After cultivation, the corresponding antibody was stained. Th1, Th17 and Treg cell populations were analyzed by flow cytometry.

For Th1 polarization, cells were cultured in RPMI-1640 cell culture medium (Gibco) (supplemented with IL-12 at 20 ng/mL and anti-IL-4 at 10 µg/mL). For Th17 polarization, cells were cultured in RPMI-1640 cell culture medium (supplemented with IL-6 at 20 ng/mL, anti-IL-4 at 10 µg/mL, anti-IFN-γ at 10 µg/mL, and TGF-β1 at 2ng/mL). For Treg polarization, cells were cultured in RPMI-1640 cell culture medium (supplemented with TGF-β1 at 5 ng/mL and IL-2 at 20 ng/mL). After 5 days of culture, the cells were collected and analyzed using FACS. Recombinant cytokines were purchased from R&D Systems (Minneapolis, USA), while antibodies against these cytokines were purchased from BD Biosciences.

### Quantification of adenosine levels using high performance liquid chromatography (HPLC)

Naive T-cells were cultured in RPMI-1640 cell culture medium supplemented with anti-CD3 and anti-CD28. After three days of culture, 50 µL cell supernatant was collected and transferred to a 1.5 ml EP tube. Subsequently, 50 µL of methanol was added, followed by thorough vortex mixing. Then, 100 µL of acetonitrile was added, vortexed for 30 s, and centrifuged at 12,000 rpm for 10 min at 4℃. The supernatant was collected for mass spectrometry detection. The subsequent performance liquid chromatography (HPLC) procedures were conducted using the ACQUITY UPLC I-Class/Xevo TQ-XS (Waters, USA). A series of adenosine standards ranging from 200 pg/ml to 20,000 pg/ml were prepared and stored at 4℃. A standard curve was constructed using the average peak area as the x-axis and adenosine concentration as the y-axis, with the regression equation calculated. The chromatographic column used was Waters BEH C18 (2.1*100 mm, 1.7 μm), maintained at 30℃.

### Statistical analysis

The data obtained from all experiments were expressed as mean ± standard deviation (mean ± SD). In contrast, the one-way analysis of variance (one-way ANOVA) was utilized determine the statistical significance. These data were analyzed using the software GraphPad Prism 9.4 (GraphPad Software, USA). The criterion for statistical significance was set at *P* < 0.05.

## Results

### Identification of mesenchymal stem cells

**Fig. 1:**
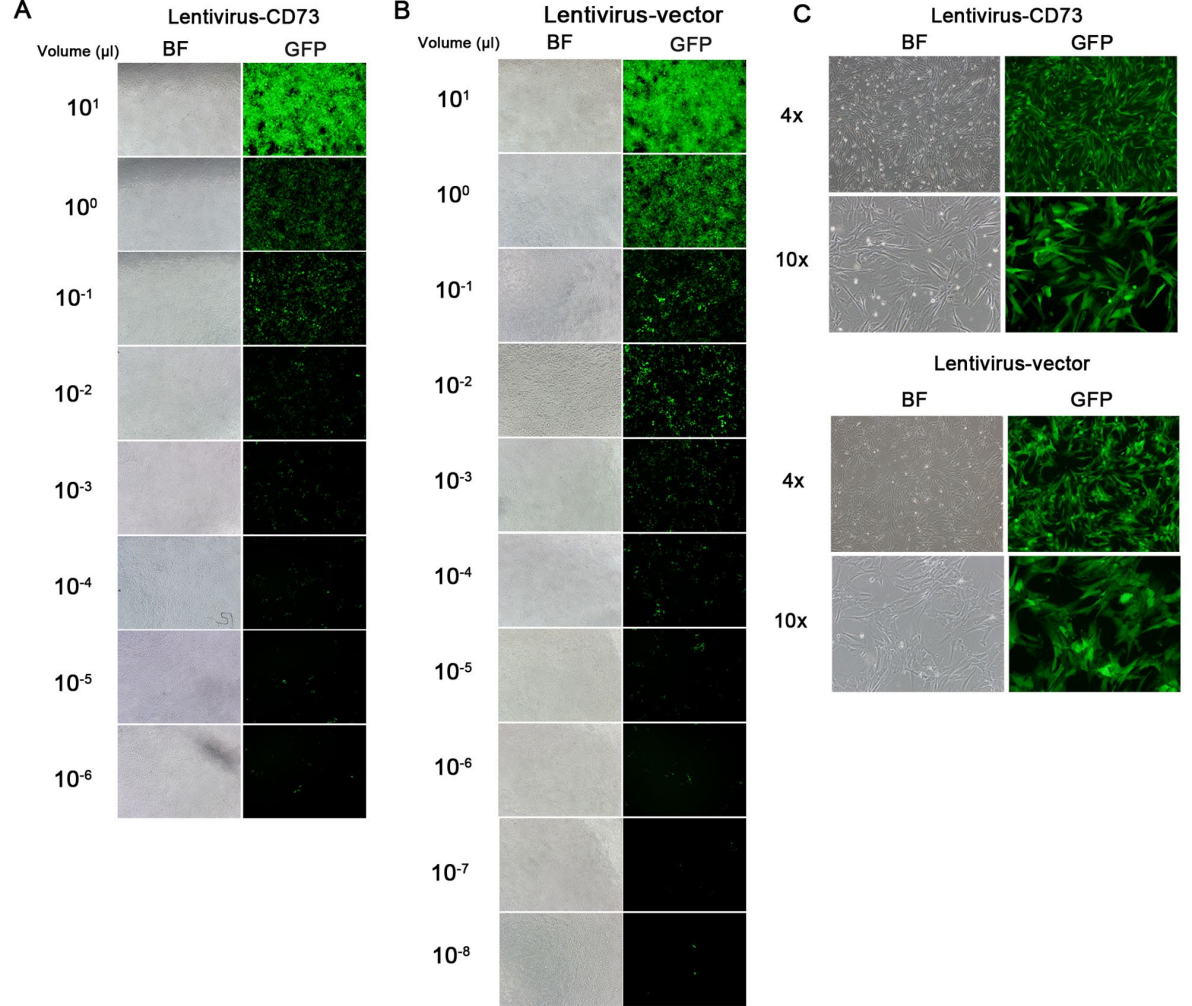
Lentiviral titration assays and transduction. (**A-B**) Lentivirus titration assays depicted the over-expression of CD73 and empty vector controls, with viral titers reaching approximately 7 × 10^7^TU/ml and 2 × 10^9^TU/ml, respectively. (**C**) Bright field (BF) and dark field images revealed targeted gene expression 48 h post-lentiviral infection of MSCs. The high expression of GFP confirmed the successful transduction of the lentivirus.

The microscopic examination revealed the spindle-shaped morphology of MSCs adhering to the culture flask walls (Fig. 1C). Furthermore, under specific differentiation conditions, MSCs demonstrated their ability to differentiate into osteocytes, chondrocytes, and adipocytes (Supplementary figure S1). Additionally, the flow cytometry identification of surface markers on MSCs can be referenced based on our previous findings [41].

### Transfection of lentivirus into mesenchymal stem cells

To obtain a high expression lentivirus vector of CD73, we packaged the constructed plasmid into HEK-293T cells and measured the titer of the resulting supernatant after concentration. We found that the transfected HEK-293 T cells exhibited high green fluorescent protein (GFP) fluorescence expression under a fluorescence microscope. The titer of the CD73-overexpressed lentivirus reached 7 × 10^7^ TU/ml, while that of the control vector was 2 × 10^9^ TU/ml (Fig. 1A, B). The MSCs were then transduced with the virus, and high fluorescence expression was observed under a fluorescence microscope 48 h post-transfection (Fig. 1C).

### Identification of mesenchymal stem cell-derived small extracellular vesicles

**Fig. 2:**
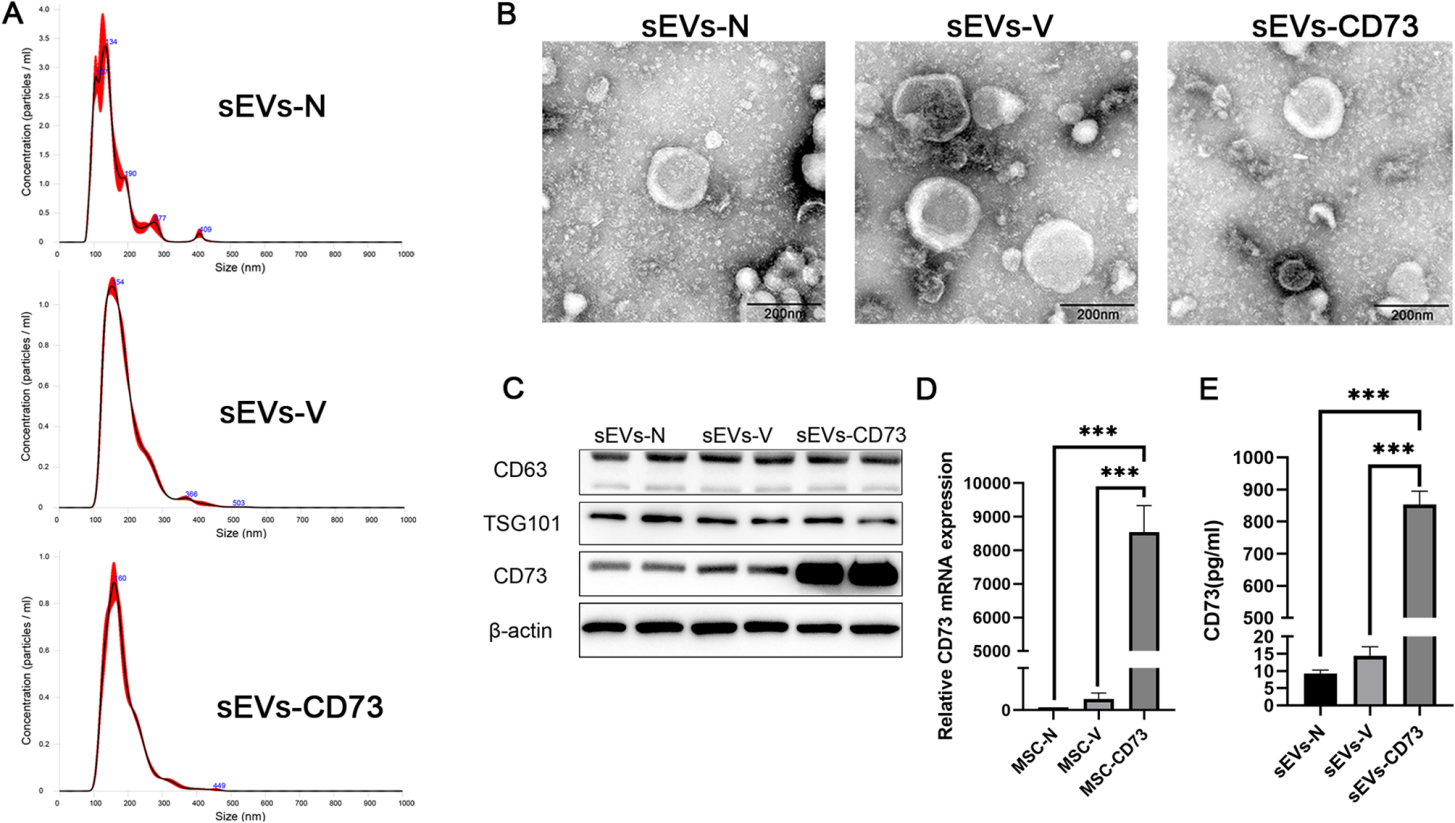
Identification of MSC-sEVs. (**A**) Nanosight analysis depicting the size distribution of various MSC-sEVs. (**B**) TEM images illustrating the morphology of various MSC-sEVs. Scale bar = 200 nm. (**C**) Western blotting results indicating the expression of CD63, TSG101, and CD73 in sEVs-N, sEVs-V, and sEVs-CD73 group. To maintain conciseness, cropping was performed. Full-length blots were presented in Supplementary Figure S2. (**D**) Real-time PCR results showing robust expression of the target gene after viral infection of MSCs. (**E**) ELISA results demonstrating elevated levels of the target protein in sEVs-CD73 compared to the other groups. Mean ± SD, *n* = 3 per group, one-way ANOVA test. ***: *P* < 0.001

Firstly, we analyzed the size and morphology of sEVs purified from the conditioned medium of MSCs using Nanosight and Transmission electron microscopy (TEM). Nanosight analysis revealed that the average diameters of sEVs-N, sEVs-V, and sEVs-CD73 were 152 nm, 189 nm, and 183 nm, respectively, consistent with our definition of sEVs (Fig. 2A). TEM images revealed that sEVs-N, sEVs-V, and sEVs-CD73 exhibited uniform size, circular shape, and double-layered membrane vesicular structures, consistent with the typical characteristics of sEVs (Fig. 2B). Additionally, TEM analysis also indicated that the diameters of all three types of sEVs were smaller than 200 nm. Western blotting results confirmed the expression of CD63, TSG101, and CD73 in all groups, with successful high expression of CD73 in the sEVs-CD73 group (Fig. 2C). Real-time PCR analysis showed high expression of CD73 mRNA in MSCs transfected with CD73-overexpressing lentivirus, consistent with the findings under fluorescence microscopy (Fig. 2D). ELISA results indicated higher levels of the target protein in sEVs-CD73 compared to the other groups (Fig. 2E). These results indicated that the vesicles isolated from MSCs exhibit characteristics consistent with sEVs and we successfully engineered sEVs overexpressing CD73.

### Augmented suppression of experimental autoimmune uveitis following intravenous administration of CD73-overexpressing mesenchymal stem cell-derived small extracellular vesicles

**Fig. 3:**
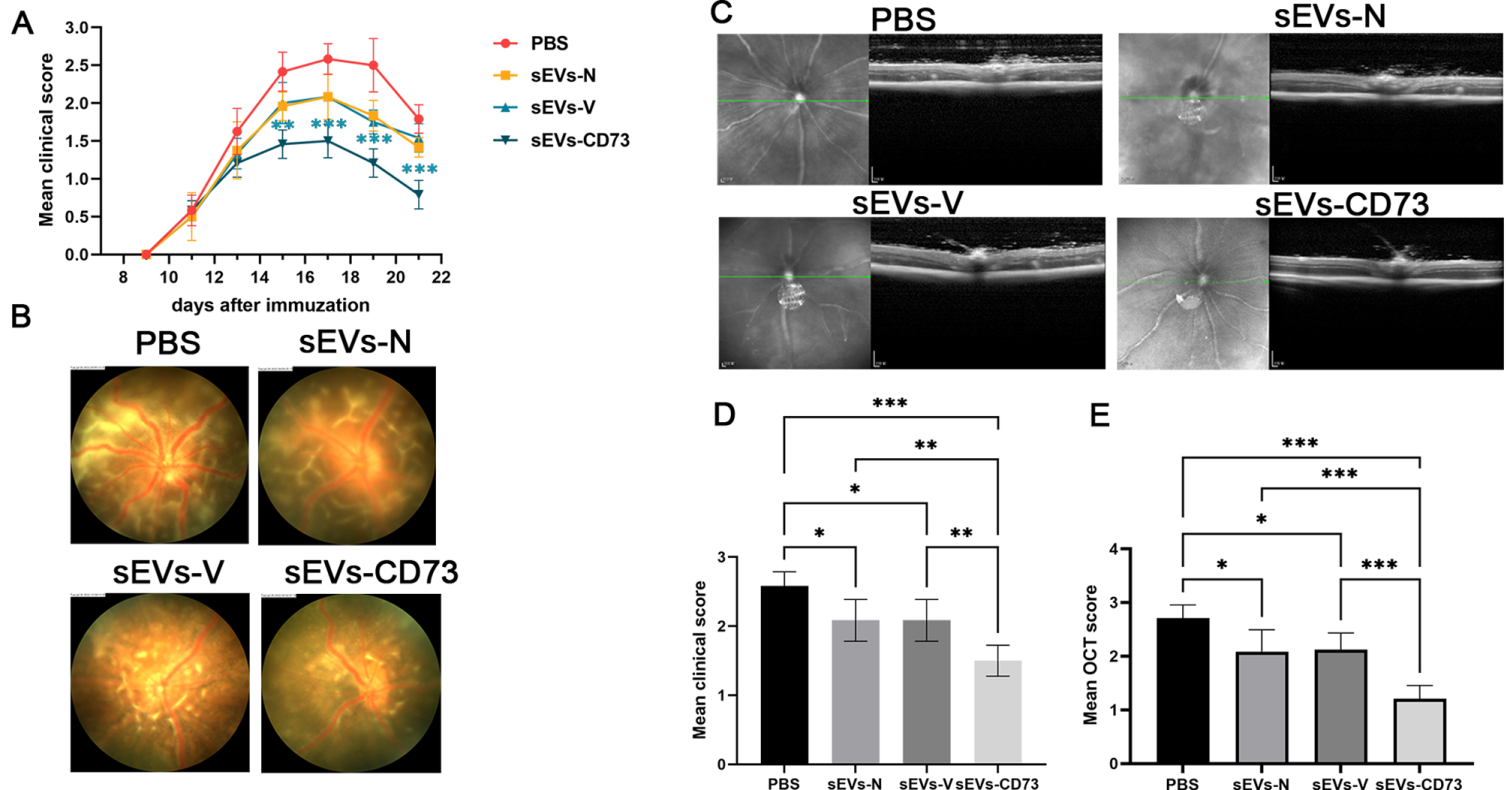
Over-expression of CD73 enhanced the therapeutic efficacy of MSC-sEVs in EAU. (**A, D**) Mean clinical scores of mice treated with tail vein injection of 50ug sEVs recorded every 2 d from day 9 to day 21 post-immunization. (**B**) The fundus imaging of each sEVs-treated group on day 17 post-immunization. (**C, E**) On day 17 post-immunization, OCT was conducted, and the outcomes were quantified and presented as OCT scores. Mean ± SD, *n* = 6 per group, one-way ANOVA test. *: *P*<0.05; **: *P*<0.01; ***: *P*<0.001

**Fig. 4:**
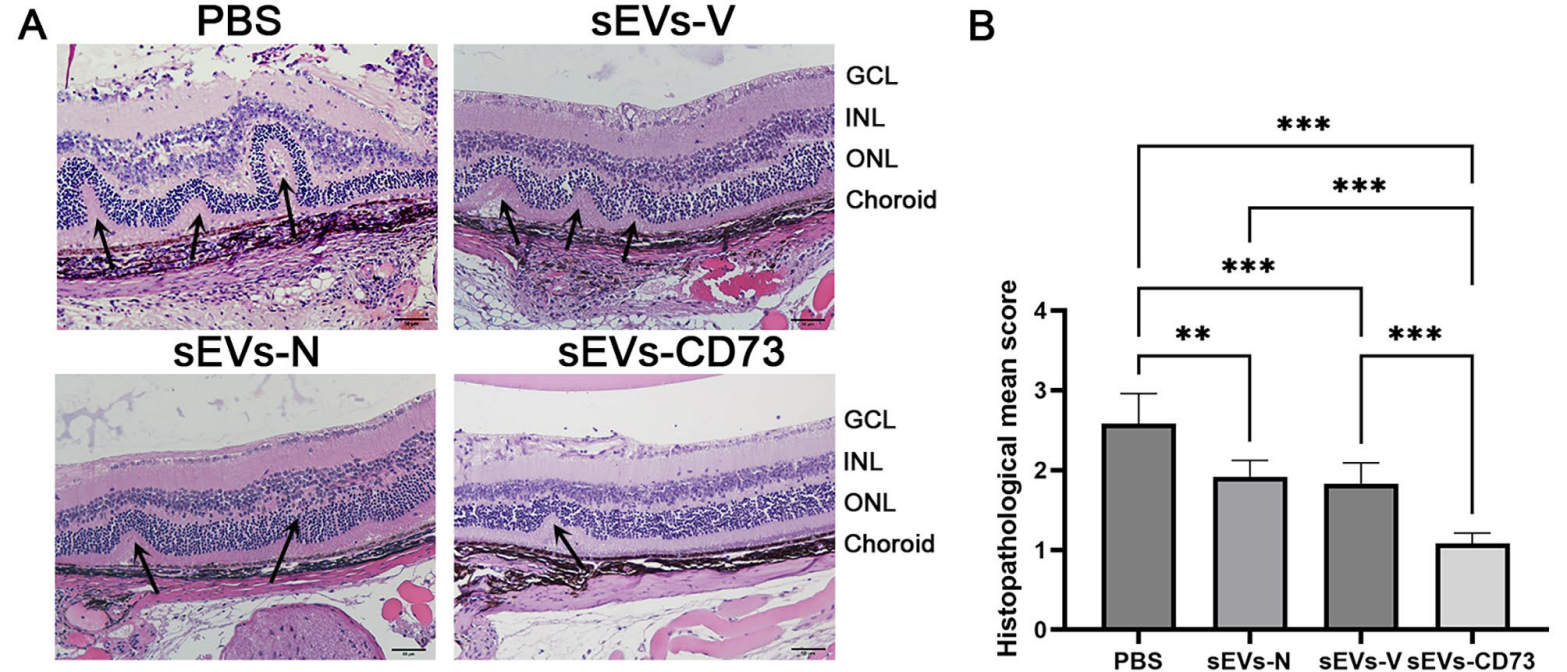
Treatment with sEVs-CD73 in EAU mice showed reduced inflammatory cell infiltration, retinal folds and granulomas. (**A**) Representative H&E-stained retinal cross-sections from different groups. The black arrows indicated retinal folds and detachments near the optic disk. GCL, ganglion cell layer; INL, inner nuclear layer; ONL, outer nuclear layer. (**B**) Quantitative histopathological scores of the retina in each group. Mean ± SD, *n* = 6 per group, one-way ANOVA test. **: *P*<0.01; ***: *P* < 0.001

To compare the therapeutic efficacy, we administered intravenous injection of 50 µg of sEVs-N, sEVs-V and sEVs-CD73 to mice on the 11th day post-immunization. The clinical scoring revealed inflammatory responses in the fundus around the 11th day post-immunization. From day 15 to day 21 post-immunization, the sEVs-CD73-treated group demonstrated a significantly lower mean clinical score compared to the other groups (*P* < 0.05) (Fig. 3A). The peak of clinical scores was observed on day 17 post-immunization. On day 17 post-immunization, the mean clinical scores of the sEVs-N and sEVs-V groups were lower than those of the PBS group, although the difference between these two groups was not statistically significant. However, both groups exhibited higher clinical scores compared to the sEVs-CD73 group (Fig. 3D). This finding was further corroborated by fundus images (Fig. 3B). OCT results also demonstrated low OCT signal and severe inflammation cell infiltration at the disease peak in the PBS group, while the sEVs-N and sEVs-V groups displayed milder inflammation. Remarkably, treatment with sEVs-CD73 exhibited the most significant inhibition of these changes among all groups (Fig. 3C). The OCT scores in the sEVs-CD73 group were significantly lower than those in the other three groups (*P* < 0.05) (Fig. 3E). In addition, histological analysis confirmed these findings, with the sEVs-CD73-treated mice exhibiting fewer infiltrating inflammatory cells, reduced retinal folds, detachment, and granulomas than the other three groups (Fig. 4). These findings suggest that overexpression of CD73 enhances the protective effect of sEV-N on the retina during ocular inflammation.

### Reduced infiltration of inflammatory cells following treatment of CD73-overexpressing mesenchymal stem cell-derived small extracellular vesicles

**Fig. 5:**
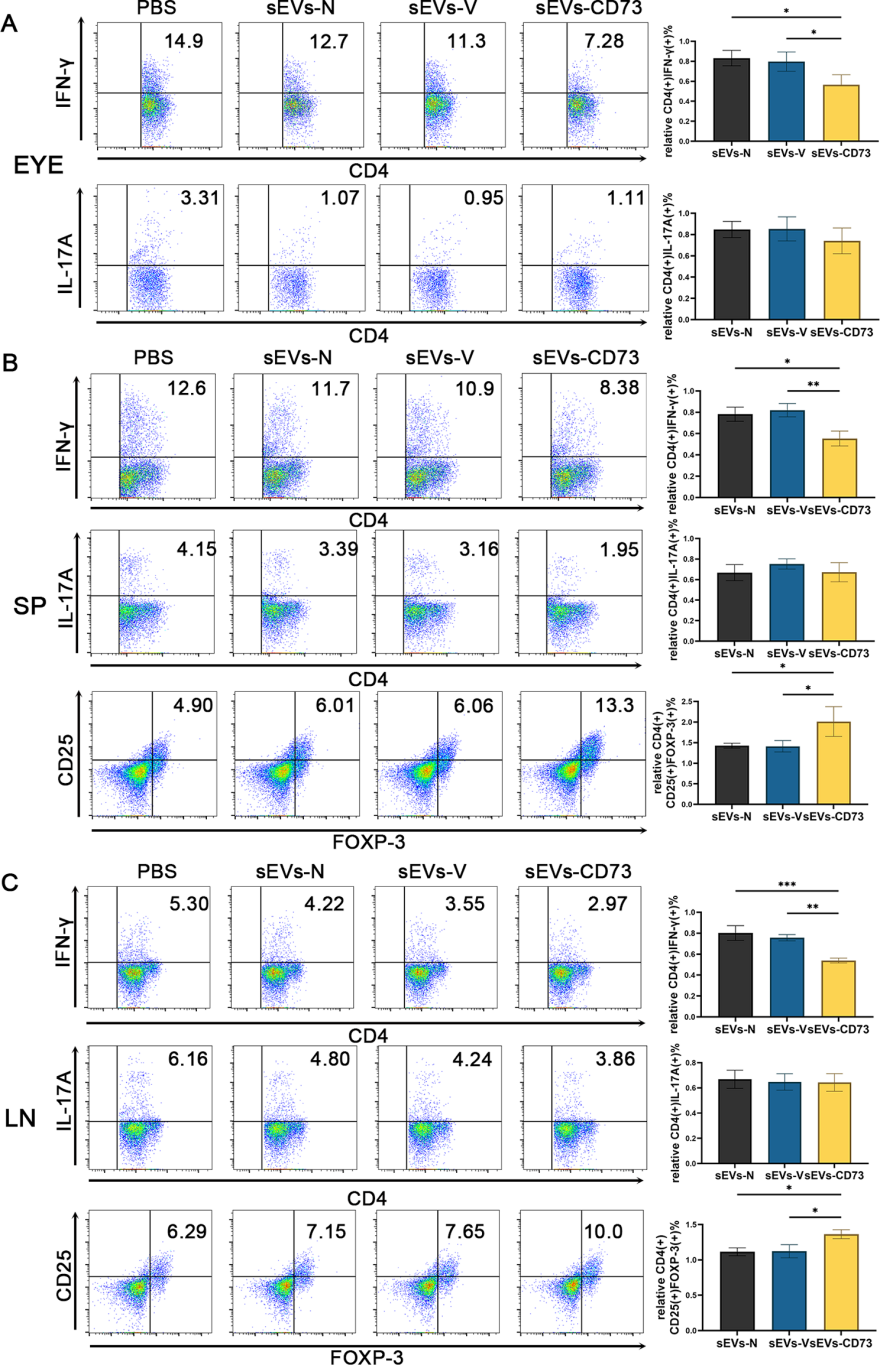
sEVs-CD73 treatment in EAU mice inhibited Th1 cells and increased Treg cells compared to sEVs-N. (**A**) Flow cytometric results of the proportions of Th1 (CD4^+^IFN-γ^+^) cells and Th17 (CD4^+^IL-17A^+^) cells in the eyeballs (*n* = 5) of mice from different groups. (**B**) Flow cytometric results of the proportions of Th1 cells, Th17 cells and Treg (CD4^+^FOXP3^+^CD25^+^) cells in the SP of mice (*n* = 6) from different groups. (**C**) Flow cytometric results of the proportions of Th1 cells, Th17 cells and Treg cells in the LNs of mice (*n* = 6) from different groups. We performed relative quantification analysis to compare the experimental groups with a blank control group, which served as the baseline. Mean ± SD, one-way ANOVA test. *: *P*<0.05; **: *P*<0.01; ***: *P*<0.001

T-cells, including Th1, Th17 and Treg cells, play crucial roles in cell-mediated immunity. In our previous studies, we observed that compared to the PBS control group, intravenous injection of 50 µg sEVs-N resulted in a lower percentage of Th1 and Th17 cells in the eyes of mice, and a higher percentage of Treg cells in the spleen and draining lymph nodes [41]. To better elucidate the influence of CD73 overexpression on sEVs, we collected tissues, including eyeballs, SP, and LNs, from mice to analyze the proportion of various T-cell subsets in each group at the peak of the disease. During the process of detecting T-cells in the eyeballs, one mouse was excluded due to death. Flow cytometric results showed a decrease in the proportions of Th1 cells (CD4^+^IFN-γ^+^) in the sEVs-CD73 group compared to the sEVs-N and sEVs-V groups in the eyeballs, SP and LNs (*P* < 0.05) (Fig. 5A-C). Surprisingly, CD73 overexpression did not significantly enhance or inhibit the effect of MSC-sEVs on Th17 cells (CD4^+^IL-17 A^+^) in these immune-related tissues (Fig. 5A-C). Moreover, the proportion of Treg cells (CD4^+^CD25^+^Foxp-3^+^) also exhibited significant changes in the SP and LNs (*P* < 0.05). As illustrated in Fig. 5B-C, the proportion of Treg cells in the sEVs-CD73 group was significantly higher than that in the sEVs-N and sEVs-V groups (*P* < 0.05). These findings suggest that MSC-sEVs exhibiting high CD73 expression possess augmented inhibitory capabilities against Th1 cells while exerting activating effects on Treg cells. However, CD73 overexpression did not significantly alter the inhibitory effects of sEVs on Th17 cells.

### Amplified inhibitory effect of CD73 over-expressing mesenchymal stem cell-derived small extracellular vesicles on T cell proliferation

**Fig. 6:**
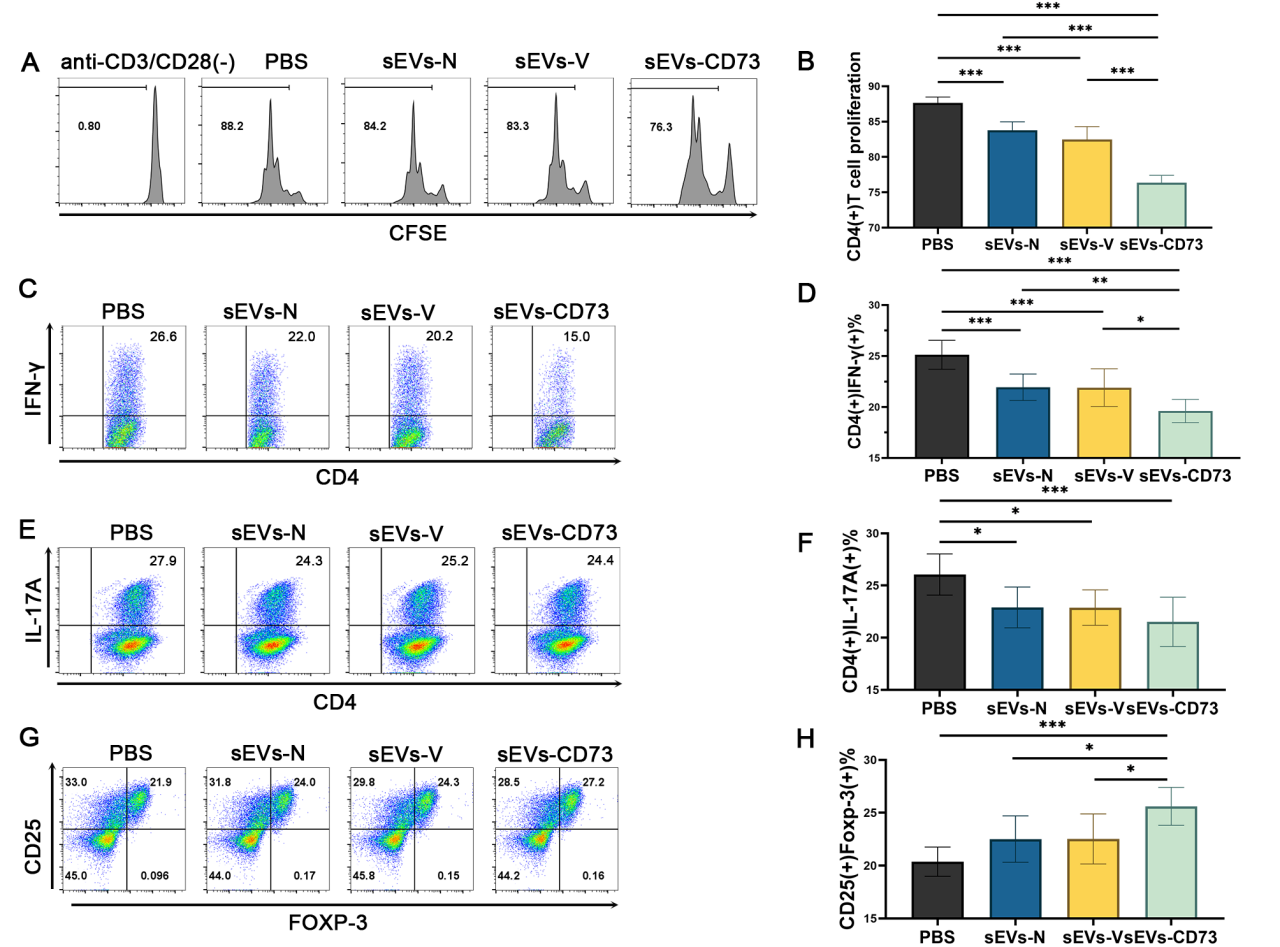
sEVs-CD73 inhibited T-cell proliferation and Th1 cell differentiation, while stimulating Treg cells in vitro. (**A, B**) In vitro inhibition of T-cell proliferation by sEVs-CD73 compared to other groups. (**C, D**) In vitro inhibition of Th1 cell differentiation by sEVs-CD73 compared to other groups. (**E, F**) In vitro unchanged effect of Th17 cell differentiation by sEVs-CD73 compared to sEVs-N and sEVs-V groups. (**G, H**) In vitro promotion of Treg cell differentiation by sEVs-CD73 compared to other groups. Mean ± SD, *n* = 3 per group, one-way ANOVA test. All experiments were independently repeated 3 times. *: *P*<0.05; **: *P*<0.01; ***: *P* < 0.001

The effect of sEVs-CD73 on T-cell proliferation was evaluated by isolating CD4^+^T-cells from SP and LNs of naïve mice using a magnetic bead positive selection kit. These T-cells were then co-cultured with MSC-sEVs from each group at various concentrations, and CFSE expression was assessed after four days of culture. Our previous studies confirmed the impact of different concentrations (0, 1, 10, 50, and 100 µg/mL) of sEVs-N on the proliferation of initial CD4^+^ T-cells. The sEVs-N group exhibited a notable inhibitory effect compared to the PBS group at a concentration of 10 µg/mL, suggesting a concentration-dependent response [41]. Consequently, we selected this concentration of 10 µg/mL for further investigation. In the current study, flow cytometric analysis revealed that sEVs-CD73 exhibited a more robust inhibitory effect on T-cell proliferation compared to the sEVs-N and sEVs-V groups at a concentration of 10 µg/mL (Fig. 6A-B). These findings indicate that overexpression of CD73 enhances the inhibitory effect of sEVs-N on T cell proliferation in vitro.

### Enhanced effect of CD73 over-expressing mesenchymal stem cell-derived small extracellular vesicles on Th1 and Treg cell differentiation

Naive T-cells combined with 10 µg/ml of various MSC-sEVs in specific differentiation conditions were cultured for 4 days to assess immune cell proportions. Compared to the other groups, the sEVs-CD73 group exhibited significantly enhanced inhibitory effects on Th1 cell (CD4^+^IFN-γ^+^) proportion (Fig. 6C, D) and promoting effects on Treg cell (CD4^+^CD25^+^Foxp-3^+^) proportion (*P* < 0.05) (Fig. 6G, H). However, there were no significant differences in the inhibitory effects on Th1 and Treg cell differentiation between the sEVs-N and sEVs-V groups (*P* > 0.05). Regarding Th17 cell (CD4^+^IL-17A^+^) differentiation, there were no significant differences among these three groups treated with sEVs (*P* > 0.05) (Fig. 6E, F). These results are consistent with findings from in vivo studies, suggesting that CD73 overexpression may enhance the immunosuppressive effects of sEVs-N by modulating the proportions of Th1 and Treg cells rather than Th17 cells.

### CD73-enriched mesenchymal stem cell-derived small extracellular vesicles increased adenosine levels in the T-cell supernatant

**Fig. 7:**
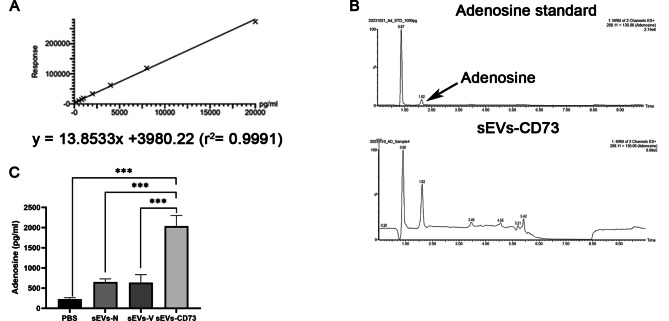
sEVs-CD73 facilitates the generation of adenosine in vitro. (**A**) The linear regression equation of adenosine standard curve. (**B**) The chromatographic peak of adenosine in sEVs-CD73 group detected by HPLC. (**C**) After co-culturing with T-cells, cell supernatant from the sEVs-CD73 group exhibited the highest proportion of secreted adenosine among the four groups. Mean ± SD, *n* = 3 per group, one-way ANOVA test. ***: *P*<0.001

Using HPLC, the adenosine content in the supernatant of Naive T-cells co-cultured with different groups of MSC-sEVs was analyzed. The linear regression equation for the adenosine standard curve was Y = 13.8533X + 3980.22, with an R^2^ value of 0.9991 (Fig. 7A). A distinct peak at 1.62 min was observed (Fig. 7B). The addition of sEVs-N and sEVs-V led to an increase in adenosine production compared to the control group, although not reaching statistical significance. However, the sEVs-CD73 group showed a significant increase in adenosine compared to the other three groups (Fig. 7C).

## Discussion

Our study established that, in comparison to the control group, MSC-sEVs exhibiting elevated CD73 expression exerted a more robust suppressive effect on the progression of EAU. In vivo experiments indicated that sEVs-CD73 can significantly reduce tissue infiltration of EAU in mice compared to normal MSC-sEVs. This alteration may be achieved by inhibiting T-cell proliferation, reducing the Th1 ratio, and increasing the Treg ratio. In vitro results were consistent with those observed in vivo. Moreover, we speculated that the suppressive effect of sEVs-CD73 on EAU was likely mediated by the increased production of adenosine.

Traditional treatment methods for uveitis typically involve the administration of corticosteroids, immunosuppressive agents, or biologic therapies. Prolonged use of corticosteroids and immunosuppressive agents can lead to ocular complications and systemic side effects. Biologic therapies, such as anti-tumor necrosis factor agents, may be expensive and require regular monitoring for adverse effects. While MSCs show promise in treating immune-related diseases like autoimmune uveitis, clinical studies have raised concerns about its safety and efficacy [34, 42, 43]. Moreover, the characteristics of MSCs are influenced by the tissue source and donor physiological status, which should be taken into consideration in clinical applications [44]. Compared to existing treatment methods, sEVs-CD73 demonstrates significant advantages. Firstly, sEVs-based therapy harnesses the therapeutic potential of EVs. After treatment, sEVs exhibit excellent biocompatibility, allowing them to penetrate the blood-retinal barriers and effectively reach target sites. Secondly, as natural vesicles, sEVs exhibit inherent safety, offering a choice for the treatment of immune disorders.

Our research team previously observed that on the 11th day post-immunization, mice treated with 50 µg MSC-sEVs via tail vein injection exhibited a reduction in the proportion of Th1 and Th17 cells compared to the control group, while the proportion of Treg cells was elevated [41]. Previous research suggested that CD73-mediated adenosine production by MSCs contributes to their immunomodulatory effects, suggesting a potential mechanism for their therapeutic efficacy in EAU [28]. This finding aligns with previous literature regarding the mechanism of action of MSC-sEVs. We hypothesized that sEVs-CD73 may regulate the Th1/Th17/Treg cell balance by promoting adenosine generation, thereby creating an immune-suppressive microenvironment. Our results indicated that the proportions of Th1 and Treg cells were consistent with our expectations. However, despite the overexpression of CD73, the administration of MSC-sEVs did not significantly alter the suppressive effect on Th17 cells. The relationship between CD73, its hydrolysis product adenosine, and Th17 cells remains incompletely understood. CD73 exhibits diverse biological functions across different cell types, with its deficiency in some cells enhancing susceptibility to autoimmune diseases, while in others, the effects are reversed.

Sun et al. demonstrated contrasting effects of adenosine on Th1 and Th17 cells, mediated by various immune cells, notably γδT cells and DCs [45]. Activation of adenosine receptors amplifies the activation of γδT cells, which serve as critical promoters of Th17 responses and significantly influences DCs differentiation. This shift in DCs differentiation favors the generation of DCs that stimulate Th17 responses over those that stimulate Th1 responses. These findings suggest that CD73-mediated hydrolysis of adenosine monophosphate (AMP) to adenosine may shift the balance towards Th17 cells, thereby exacerbating autoimmune responses. During various stages of EAU, γδT cells exhibited varying levels of CD73 and A2AR expression. In active disease stages, adenosine collaborates with elevated cytokine levels, resulting in amplified activation of γδT cells and Th17 responses [46]. Upon activation, γδT cells exhibit elevated expression of A2AR but reduced levels of CD73. While heightened A2AR expression enables activated γδT cells to efficiently bind adenosine compared to other immune cells, diminished CD73 expression limits their capacity to convert AMP into adenosine, potentially exacerbating inflammatory reactions [47–49]. However, during inactive disease stages characterized by low cytokine levels, adenosine does not augment γδT cell activation. This underscores the variable activation status of γδT cells and A2AR expression during different EAU stages, influencing disease progression. The A2AR antagonist SCH significantly impeded the progression of EAU primarily by regulating Th17 responses. Nevertheless, the timing of treatment administration is crucial. The antagonist effectively curbed EAU advancement only when administered during the active disease stage, but proved ineffective if initiated during the disease induction phase [46].

In line with our findings, Hernandez-Mir and colleagues (2017) reported similar results [50]. Their results demonstrated that CD73 deficiency did not significantly influence the progression of experimental autoimmune encephalomyelitis (EAE) or the differentiation of Th17 cells in vitro. These findings contrast with those of Mills et al., who reported reduced severity of EAE in CD73-/- mice along with decreased IFN-γ secretion [51]. Discrepancies in EAE induction protocols across different laboratories may contribute to variations in the balance between Th17 and Th1 cell populations induced, thus influencing the impact of CD73 presence on disease severity. Hernandez-Mir et al. suggested a primary association between EAE and Th17 cells, while Mills et al. focused their investigation on Th1 cells, proposing a potential pivotal role of CD73 in disease development. Additionally, Hernandez-Mir et al. found that the lack of CD73 had no significant effect on the proportion of Treg cells or their recruitment to the central nervous system. In contrast, our study suggested that overexpression of CD73 enhances the promotive effect of MSC-sEVs on Treg cells in EAU-induced mice. The differences in these results may be attributed to variations in experimental models and procedural differences among different laboratories, leading to variations in the proportions of Treg and Th17 cells within the disease models. While conflicting data exists for the role of Treg cells in EAE [52, 53], they have been shown to act as inhibitory T cells in EAU. These regulatory T cells can inhibit cytokine production and T cell proliferation, and one of their immunomodulatory mechanisms involves promoting adenosine generation through CD39 and CD73, leading to increased intracellular cAMP levels upon binding to adenosine receptors. Further investigation revealed that in microenvironments enriched with sEVs expressing CD73 or other CD73-expressing lymphocytes, CD4^+^CD39^+^Tregs readily interact with surface CD73, mediating adenosine-driven immunosuppressive effects [54]. Although our results demonstrate that MSC-sEVs overexpressing CD73 cannot directly inhibit the differentiation of Th17 cells, these vesicles effectively suppress Th1 cells and promote Treg cells. Given the critical roles of Th1 and Treg cells in the development of EAU, sEVs-CD73 can shift the balance of immune-related cells towards Treg cells, resulting in more potent immunosuppressive effects than MSC-sEVs alone, thus inhibiting the progression of EAU.

However, certain limitations and avenues for further exploration should be noted. Firstly, technical and time constraints prevented us from measuring pro-inflammatory and anti-inflammatory factors (such as IL-6, IL-4, and IL-10) in the vitreous humor of EAU mice. Additionally, we lack detection of Treg cell proportions in the eyes among all groups. These omissions may have hindered a comprehensive understanding of how our engineered sEVs modulate the immune response. Furthermore, discrepancies between our findings regarding Th17 cells and those reported in existing literature underscore the complexity of the immune response in uveitis. These inconsistencies warrant further investigation to clarify the underlying mechanisms governing Th17 cell modulation by sEVs-CD73. Moreover, the lack of safety validation is a notable concern. Safety validation is crucial for clinical translation. In summary, addressing these limitations through further research will be crucial for advancing our understanding of the safety and efficacy of CD73-overexpressed MSC-sEVs for the treatment of autoimmune uveitis.

## Conclusions

Overall, we successfully constructed MSC-sEVs with high CD73 expression. We found that these vesicles exhibited a significantly greater inhibitory effect on EAU compared to normal MSC-sEVs. This effect may be attributed to the enhancement of extracellular adenosine production, thereby augmenting the impact of MSC-sEVs on Th1 and Treg cells. Therefore, CD73-overexpressing MSC-sEVs could present a novel therapeutic avenue for addressing autoimmune uveitis or other autoimmune disorders.

### Electronic supplementary material

Below is the link to the electronic supplementary material.

**Additional file 1**: Figure S1. Identification of MSCs. Specific differentiation conditions promote successful differentiation of MSCs into osteoblasts, chondrocytes, and adipocytes.

**Additional file 2**: Figure S2. The Western blot gel image depicted protein expression patterns in sEVs-N, sEVs-V, sEVs-CD73. To minimize cross-contamination between different antibodies, two gels were prepared to detect the expression of surface proteins on distinct groups of sEVs. Gel 1 was utilized for detecting CD63 and TSG101, while Gel 2 was employed for incubating with CD73. Both gels were loaded with the same amounts of samples, and β-actin was probed on each gel to ensure consistency in loading. Lane assignments were as follows: Lane 1, 2 (sEVs-N); Lane 3, 4 (sEVs-V); Lane 5, 6 (sEVs-CD73). For conciseness, the images were cropped, as indicated by the boxed area.

**Additional file 3**: Figure S3. Flow cytometry gating strategy for Th1 and Th17 cells in the eye, spleen, and lymph nodes. Cell suspensions were prepared from eye (A), spleen (B), and lymph node tissues (C), and stained with Brilliant Violet™ 711 anti-mouse CD4, FITC anti-mouse IFN-γ and PE anti-mouse IL-17A antibodies. Lymphocytes were gated first, followed by selection of CD4^+^T-lymphocytes, and subsequently gated for specific IFN-γ and IL-17A expression.

## Data Availability

The statistical numeric data and figures supporting the conclusions of this article are openly available in Figshare repository at figshare.com (10.6084/m9.figshare.25709115). Additional data are available from the corresponding author upon reasonable request.

## Abbreviations

AMP: adenosine monophosphate
ANOVA: analysis of variance
ARVO: Association for Research in Vision and Ophthalmology
ATP: adenosine triphosphate
BF: bright field
CFA: complete Freund’s adjuvant
CFSE: carboxyfluorescein diacetate succinimidyl ester
EAE: experimental autoimmune encephalomyelitis
EAU: experimental autoimmune uveitis
FBS: fetal bovine serum
GCs: glucocorticoids
GFP: green fluorescent protein
HEK-293 T: human embryonic kidney 293 T cells
IRBP: interphotoreceptor retinoid-binding protein
H&E: hematoxylin and eosin
HPLC: liquid chromatography
ISCT: International Society for Cell & Gene Therapy
LN: lymph nodes
MISEV: Minimal information for studies of extracellular vesicles
MOI: multiplicity of infection
MSCs: mesenchymal stem cells
MSC-N: normal MSCs
MSC-sEVs: mesenchymal stem cell-derived small extracellular vesicles
MSC-V: vector-infected MSCs
MSC-CD73: CD73-overexpressed MSCs
NTA: nanoparticle tracking analysis
OCT: spectralis optical coherence tomography
PD-1: programmed cell death protein 1
PKA: protein kinase A
PTX: pertussis toxin
PVDF: polyvinylidene difluoride
qRT-PCR: quantitative real-time PCR
SD: standard deviation
sEVs: small extracellular vesicles
sEVs-CD73: CD73-overexpressed MSC-sEVs
sEVs-N: normal MSC-sEVs
sEVs-V: vector-infected MSC-sEVs
SP: spleens
SPF: specific pathogen-free
TB: mycobacterium tuberculosis
TMUEC: Tianjin Medical University Eye Hospital
Treg: Regulatory T-cells
