# Supplementary figures and images for: Enhanced immunosuppressive capability of mesenchymal stem cell-derived small extracellular vesicles with high expression of CD73 in experimental autoimmune uveitis
Source: Stem Cell Res Ther. 2024 May 23;15:149. doi: 10.1186/s13287-024-03764-7 (PMC11118760; doi:10.1186/s13287-024-03764-7)

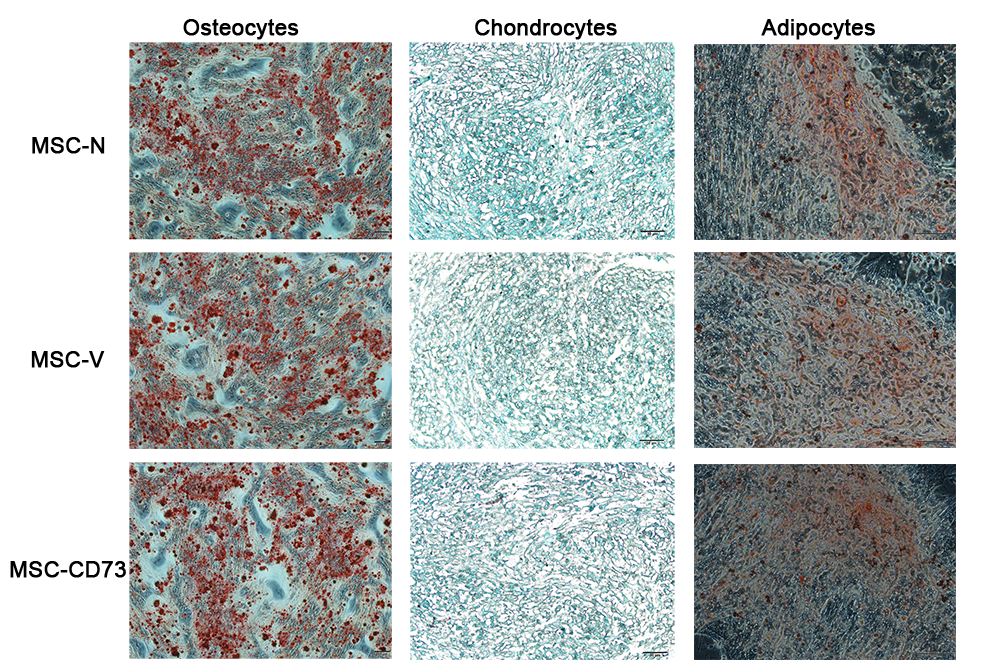

Supplement: Supplementary file 1 — Additional file 1: Figure S1. Identification of MSCs. Specific differentiation conditions promote successful differentiation of MSCs into osteoblasts, chondrocytes, and adipocytes. [file 13287_2024_3764_MOESM1_ESM.bmp]

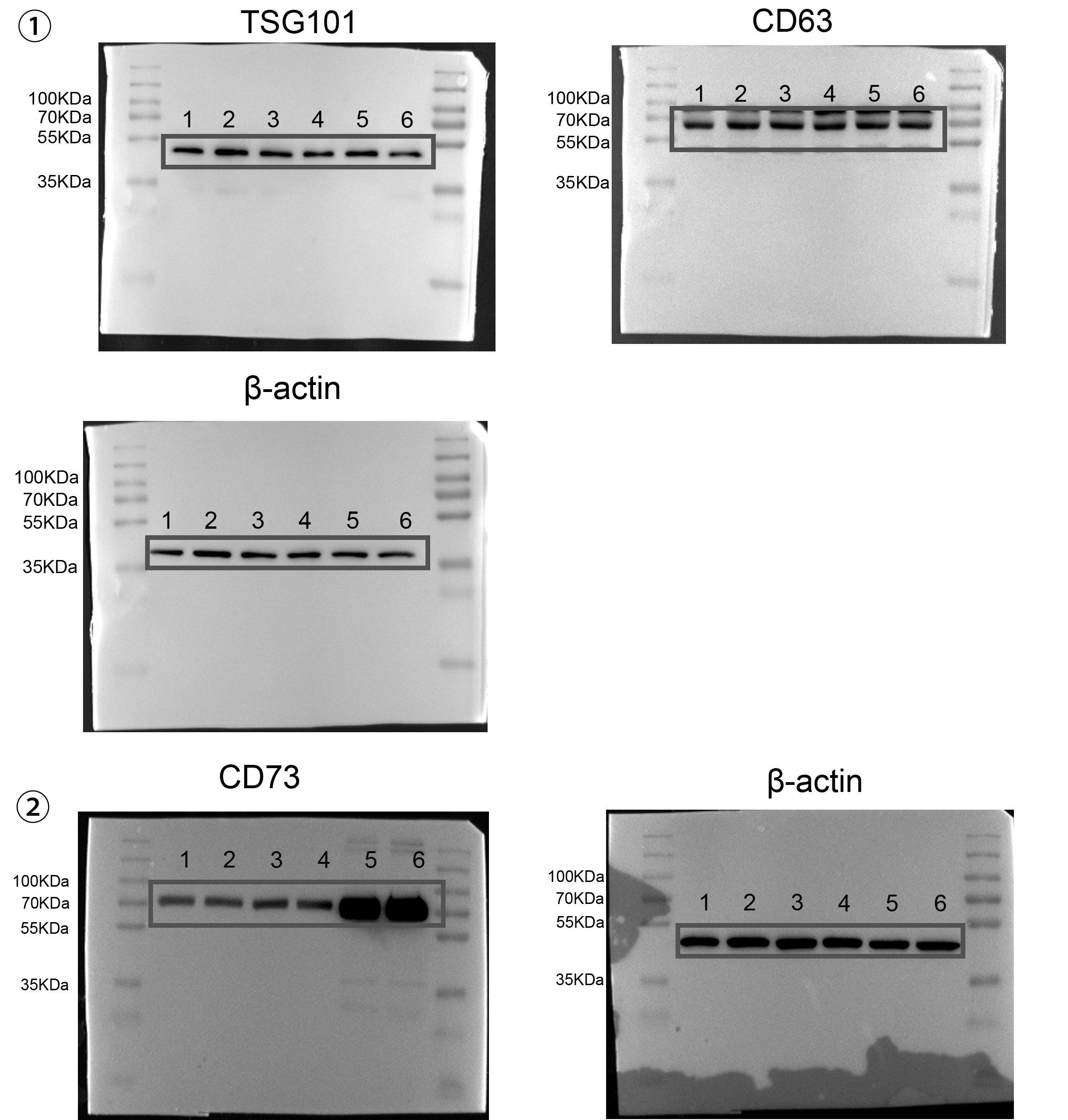

Supplement: Supplementary file 2 — Additional file 2: Figure S2. The Western blot gel image depicted protein expression patterns in sEVs-N, sEVs-V, sEVs-CD73. To minimize cross-contamination between different antibodies, two gels were prepared to detect the expression of surface proteins on distinct groups of sEVs. Gel 1 was utilized for detecting CD63 and TSG101, while Gel 2 was employed for incubating with CD73. Both gels were loaded with the same amounts of samples, and β-actin was probed on each gel to ensure consistency in loading. Lane assignments were as follows: Lane 1, 2 (sEVs-N); Lane 3, 4 (sEVs-V); Lane 5, 6 (sEVs-CD73). For conciseness, the images were cropped, as indicated by the boxed area. [file 13287_2024_3764_MOESM2_ESM.bmp]

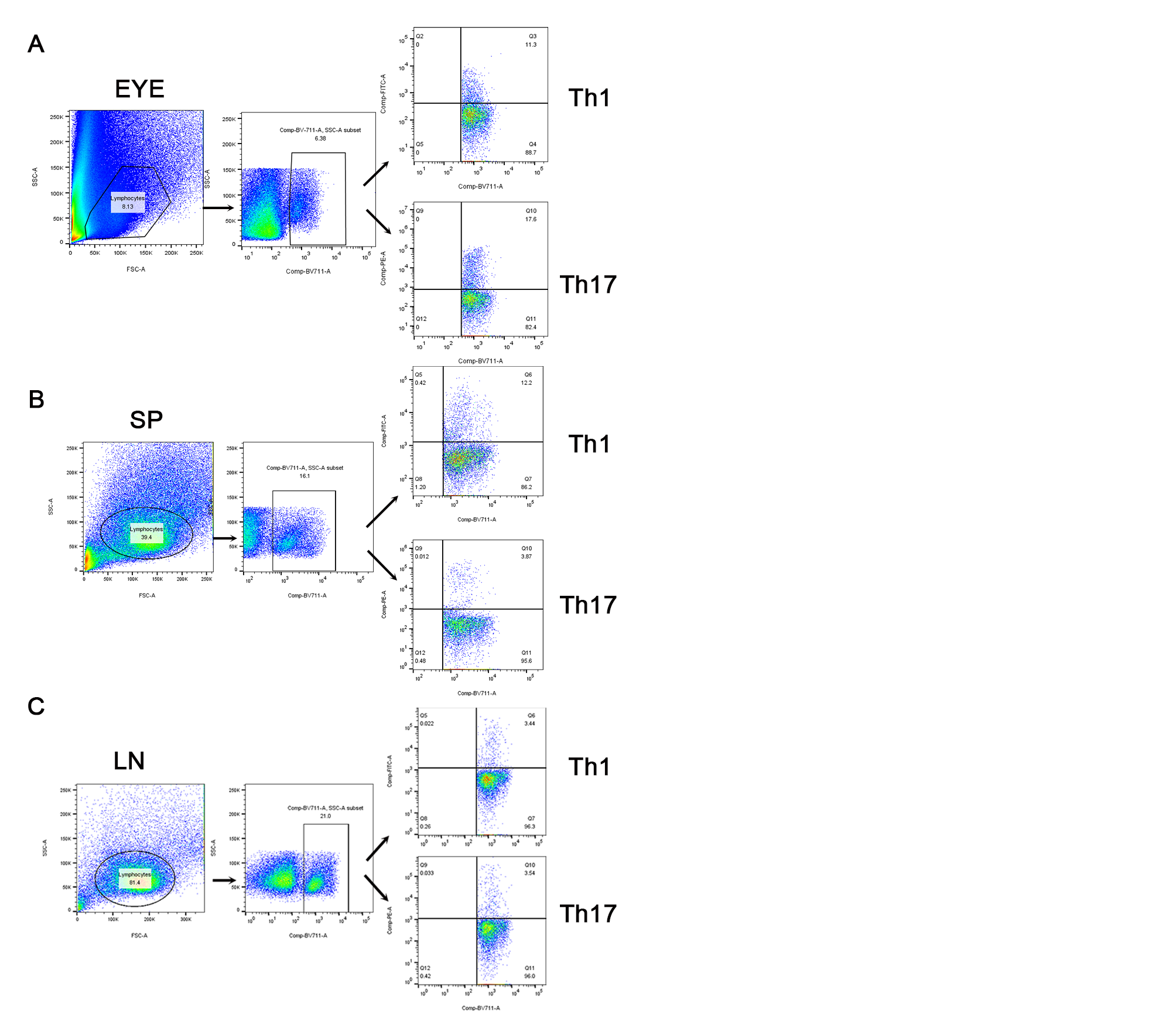

Supplement: Supplementary file 3 — Additional file 3: Figure S3. Flow cytometry gating strategy for Th1 and Th17 cells in the eye, spleen, and lymph nodes. Cell suspensions were prepared from eye (A), spleen (B), and lymph node tissues (C), and stained with Brilliant Violet™ 711 anti-mouse CD4, FITC anti-mouse IFN-γ and PE anti-mouse IL-17A antibodies. Lymphocytes were gated first, followed by selection of CD4+T-lymphocytes, and subsequently gated for specific IFN-γ and IL-17A expression. [file 13287_2024_3764_MOESM3_ESM.bmp]
